# Food and Drug Administration Expert Panel on Infant Formula “Operation Stork Speed” June 2025: Part 2, Regulatory and Safety Considerations^☆^

**DOI:** 10.1016/j.advnut.2025.100584

**Published:** 2026-01-12

**Authors:** Steven A Abrams, James Thomas Brenna, Roger Clemens, Valeria C Cohran, Nan Du, Andrea Gilbaugh, Michael I Goran, Analeise Guild, John A Kerner, Thomas B Knudsen, Sushma Krishna, Timothy Sentongo

**Affiliations:** 1 Department of Pediatrics, Dell Medical School at the University of Texas, Austin, TX, United States; 2 Department of Regulatory and Quality Sciences, USC Mann School of Pharmacy and Pharmaceutical Sciences, University of Southern California, Los Angeles, CA, United States; 3 Department of Pediatrics, The Ann & Robert H. Lurie Children’s Hospital of Chicago, Feinberg School of Medicine, Chicago, IL, United States; 4 Center for Nutrition, Division of Gastroenterology, Hepatology, and Nutrition, Boston Children's Hospital, Boston, MA, United States; 5 Clinical Nutrition, Stanford Medicine Children's Health, Palo Alto, CA, United States; 6 Department of Pediatrics, Children’s Hospital Los Angeles and Keck School of Medicine, University of Southern California, Los Angeles, CA, United States; 7 Global Gastroschisis Foundation, Wake Forest, NC, United States; 8 Division of Gastroenterology, Hepatology, and Nutrition and Division of Neonatology and Developmental Medicine, Stanford University Medical Center, Palo Alto, CA, United States; 9 Department of Intelligent Systems Engineering, Indiana University, Bloomington, IN, United States; 10 Division of Neonatology, New York-Presbyterian – Weill Cornell Medicine, New York, NY, United States; 11 Department of Pediatrics, Pritzker School of Medicine, University of Chicago, Chicago, IL, United States

**Keywords:** infant formula, generally recognized as safe, heavy metals, formula safety, infant nutrition

## Abstract

Operation Stork Speed was launched to modernize infant formula oversight after 2022 shortages and other evidence of supply chain and safety issues. Current Food and Drug Administration (FDA) processes to regulate formulas are at times slow and complex, making it difficult for new formulas to enter the market. One key pathway to adding bioactive substances or other compounds to infant formula is via the generally recognized as safe (GRAS) route. GRAS and food additive pathways require safety data, but food additive petitions require more safety information and cannot be marketed until FDA approval is granted. Concern has been expressed about the safety of the formula related to the possible presence of toxic substances in the formula. Heavy metals, perfluoroalkyl and polyfluoroalkyl substances, and other toxins can be found in formulas, and infants can be at increased risk of effects. United States lacks enforceable limits, unlike the European Union, Canadian, and Australian counterparts. To enhance the regulatory environment for infant formula, legislative updates, supply chain transparency, and alignment with global safety standards are needed.

**Statement of Significance**

Infant formula is highly regulated in the United States. This manuscript discusses an expert panel conference related to this topic held at the Food and Drug Administration in June 2025.

## Introduction: Regulation of Infant Formula and Operation Stork Speed

Operation Stork Speed, a joint initiative between the United States Department of Health and Human Services and the United States Food and Drug Administration (FDA), was announced in March 2025. The FDA requested that an expert group be convened to discuss key issues and establish a plan for a meeting to be held at the FDA. The transcript of the entire meeting is publicly available [1]. Evaluation of regulatory and safety aspects of infant formula was a key part of Operation Stork Speed and aspects related to this part of the meeting are presented in this article.

## Regulatory Processes for New Formulas

Regulations around infant formula were lacking until the establishment of the Infant Formula Act of 1980, the regulatory framework governing the composition of infant formulas in the United States. Although the Infant Formula Act describes aspects of production, content, and regulatory review of infant formulas, specific guidance is provided by a series of documents from the FDA. Most of the guidance was proposed in 1996 [1,2]. In 2014, the FDA established 2 main quality factors for infant formula including normal physical growth and sufficient biological quality of protein component. Generally, the process of registering novel formulas or major changes to existing formula composition and processing conditions is somewhat cumbersome, time consuming, and expensive for companies. As such, it can hinder the introduction of new formulas to the market [1,2]. Infant formula companies new to the United States market may also be limited by the market structure shaped by the government contracting policies, such as Special Supplemental Program for Women, Infant's, and Children (WIC) Program’s sole source contracting system and costly initial capital investment required for FDA approval. They may also be limited by hospital contracting for infant formulas, which are often based on the hospital nearly exclusively using the contracted formula. The formula shortages of 2022 provided a stark example of the problem, with a limited number of formula options registered by the FDA, leading to efforts to both prevent such shortages from recurring directly and improve the registration process.

Congress requested that the National Academies of Sciences, Engineering, and Medicine (NASEM) prepare several reports to assess the supply and Regulation of Infant Formula in the United States. The first of these was published in 2024 and consists of a detailed description of recommendations for resiliency in the supply chain [2]. Critical aspects of the recommendations in this report include providing the FDA with timely information about potential product-related issues, improving the identification of registered products, and enhancing caregivers' and the public's knowledge regarding infant formula issues, so that switching brands when one is in short supply can be done more readily.

The FDA responded to the shortages and the panel with several documents providing plans to improve resiliency in the formula supply chain [3]. Certain aspects of this response will require legislation, especially those that would mandate companies to report all positive test results, not just those released into the market, and have remote access to all test data. This key regulation would provide a significant level of additional flexibility for FDA factory inspection planning and supervision. Additionally, Congressional action would assist in meeting the NASEM panel recommendation that “Congress should amend the Food, Drug, and Cosmetic Act to require manufacturers of critical foods to give sufficient advanced notice to the FDA when they decide to discontinue a critical food.”

Regarding new formulas, the FDA process focuses on protein quantity and quality studies and growth studies. The NASEM panel on this topic released its report in May 2025 [4]. Key limitations were identified in current approaches, such as cost and difficulty in interpreting results with novel protein sources, as well as limitations in recruitment of patients for clinical studies, with strong recommendations to revise the animal protein quality study and to simplify and clarify aspects of the growth study.

It is surprising to many that there is no single list of FDA-registered infant formulas. When shortages occur, some products may be considered registered but are not, such as certain toddler products and others. The 2024 NASEM [2] report recommended that “The Food and Drug Administration should maintain a public list of all infant formulas currently marketed and registered in the United States, indexing infant formula name, whether the formula is exempt or non-exempt, and the registered list of ingredients that appear on the label.”

Another aspect that may be considered in this area is whether a fully formula-fed group is always needed in clinical trials of a new formula. Comparisons may be made with the established Center for Disease Control (CDC)/WHO global infant growth standards, and a routine formula comparison may have little need and considerably restrict the conduct of such studies. Limiting enrollment to infants who are fully formula-fed by 14 d of age is another current standard not required in Europe that should be reassessed [4]. In addition, consideration should be given to methods for assessing growth and providing guidance for mixed-fed infants (those receiving both human milk and infant formula), because this represents a substantial number of infants for whom there is minimal research or specific guidance on formula use.

## Safety Components and Generally Recognized as Safe Process

The safety of all ingredients used in foods is paramount. The legal definition of ingredient safety in the United States is codified in the Code of Federal Regulations [21 CFR 170.3(i) and 21 CFR 570.3(i)]. These regulations stipulate that, based on the preponderance of scientific evidence, there is a reasonable certainty of no harm under the conditions of use. Thus, the final safety of a food ingredient ultimately depends on balancing its potential benefits and risks.

**FIGURE 1:**
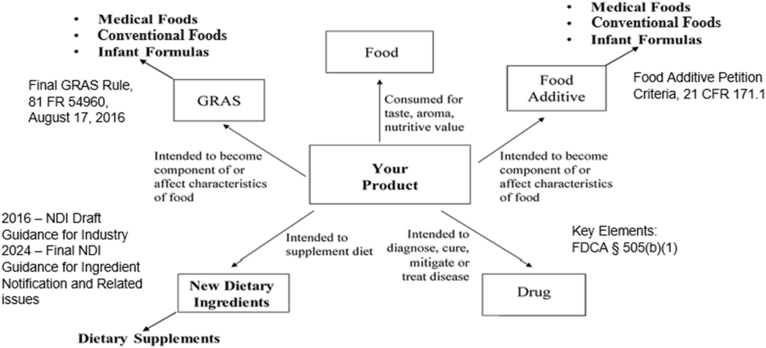
Comparison of food additive petition and GRAS process. CFR, Code of Federal Regulations; FDCA, Food, Drug, and Cosmetic Act; FR, Federal Register; GRAS, generally recognized as safe; NDI, new dietary ingredient. Adapted from the Spherix Consulting Group.

All ingredients used in the design and formulation of infant formulas intended to be marketed in the United States must adhere to the safety criteria for food additives or the generally recognized as safe (GRAS) process (Figure 1). The requirements for food additive petitions are stipulated in a 2009 FDA guidance document [5]. The final rule for GRAS was published in 2016 [6]. This action reflected, in part, the increased incorporation of novel ingredients in the food supply since the introduction of the Food Additives Amendment to the Food, Drug & Cosmetic (FD&C) Act in 1958.

Food additives introduced to the United States market after 1958, as stipulated by Congress, must undergo premarket approval. Yet, the FD&C amendment noted that the agency had not reviewed some food ingredients. In 1969, the United States government initiated the evaluation of GRAS substances, a process that commenced in 1972. The GRAS notification process began in 1997.

Although many argue that the GRAS affirmation process is biased by sponsor involvement and by the panel of scientific experts, it is essential to acknowledge a conflict of interest (COI) declaration by prospective GRAS panel members and the absence of sponsor involvement (other than providing pertinent data) in the GRAS panel review. The COI issue was discussed in a 2009 Institute of Medicine report [7] and addressed in the 2016 final ruling for GRAS [6]. This COI notice is intended to ensure that the GRAS panel members are independent and are devoid of any relationship with the GRAS sponsor and related companies associated with the food ingredient under review.

**FIGURE 2:**
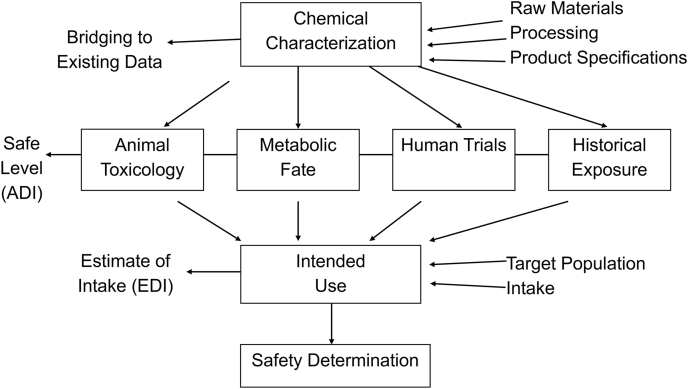
Fundamental process for ingredient safety assessment. ADI, acceptable daily intake; EDI, estimated daily intake.

The criteria for assessing ingredient safety and toxicology are identical for food additives and GRAS ingredients (Figure 2). The only differences are the time for review and potential approval, as well as the data required for the intended technical effect. For a food additive, the FDA mandates a 120-d review period. Importantly, the agency must act on a petition within 180 d. Similarly, the FDA must respond to a GRAS notification within 180 d. On the other hand, with a GRAS affirmation dossier approved by the panel of experts, the reviewed food ingredient can be immediately introduced into commercial distribution. Regarding ingredients intended for infant formula, critical clinical data would have been included in the GRAS dossier. Although a food additive petition and GRAS dossier require the same basic information (Table 1), some investigators suggest that the food additive petition requires more safety information.

**TABLE 1:** Key elements of GRAS dossier and food additive petition.

GRAS Dossier (81 FR 54960, 2016)	Food Additive Petition (21 CFR 171.1)
• Substance identification, including physicochemical properties and technical effect(s)	• Chemical identification, including physical properties and technical effect(s)
• Manufacturing process (consistent with cGMP and HACCP)	• Manufacturing process (consistent with cGMP and HACCP)
• Substance quality specifications [e.g., testing reports, heavy metal analysis, microbiology, mycotoxins, environmental toxins, toxicology (acute, chronic, mutagenicity, carcinogenicity based on animal studies and relative biological models, including clinical studies), physical properties] • Validated analytical method(s) for detecting and quantifying the substance in finished products • Quality data based on 3 nonconsecutive product batches • Substance stability assessment	• Safety assessment, including toxicology [e.g., testing reports, heavy metal analysis, microbiology, mycotoxins, environmental toxins, toxicology (acute, chronic, mutagenicity, carcinogenicity based on animal studies and relative biological models, including clinical studies)] • Stability assessment • Analytical methods for detecting and quantifying the substance in finished products
• Intended population and maximum use EDI in specific product types (based on ADI); typically based on the most recent NHANES data	• Proposed uses (population), including maximum use (based on ADI) (e.g., tolerance) and exposure (EDI); typically based on the most recent NHANES data
• Expert panel conclusions	• Environmental assessment
	• Labeling information
	• Sponsor conclusions on substance safety
	• Dossier submitted to the FDA

Abbreviations: ADI, acceptable daily intake; cGMP, current good manufacturing practices; EDI, estimated daily intake; FDA, Food and Drug Administration; GRAS, generally recognized as safe; HACCP, hazard analysis critical control point.

Some drivers of improving food safety include plant-based toxins, plant-based viruses, potential contamination of medicinal products, environmental pollutants, and biological systems that involve gastrointestinal, neurologic, and immunologic functions [8]. Although food additive petitions include a spectrum of toxicologic assessment data, there is a virtual absence of toxicologic data on potential chemical changes in these substances after specific thermal and nonthermal processing in a food matrix, and under controlled storage and shipment conditions of a finished product [9].

Interestingly, a food additive petition requires premarket approval by the FDA, whereas a GRAS affirmation process does not require FDA notification. This latter point is an issue for some organizations that contend the GRAS affirmation is a GRAS loophole that could compromise food safety [10].

Within the chemical characterization step, physicochemical properties and technical functional effects are assessed using current analytical technologies. This step considers potential processing impurities, environmental contaminants, biological toxins, and heavy metals, such as lead, mercury, cadmium, and arsenic. Information available in the public scientific literature and regulatory agencies is leveraged in this process. Traditional toxicologic evaluations typically include acute and chronic studies involving appropriate animal models plus mutagenicity and carcinogenicity using in vitro, in vivo assays and rodent bioassays. These evaluations are presented in the updated FDA Red Book (FDA) [11] and outlined in the scientific International Council for Harmonization (ICH) guidelines [12].

The ICH guidelines consider impurities, dose selection for carcinogenicity studies, genotoxicity, toxicokinetics, reproductive toxicity, and immunotoxicity. Many companies follow Organization for Economic Co-operation and Development guidelines for toxicological assessments [13]. These types of toxicologic studies in practice lead to the establishment of a no-observed-adverse-effect level (NOAEL). A typical margin of safety (MoS) is then applied to NOAEL to establish a dose considered safe for humans. A typical MoS value is 100, thereby providing a dose of a food ingredient that does not exceed health thresholds (e.g., nongenotoxic and noncarcinogenic). This dose, NOAEL/100, is often expressed as mg/kg body weight per day (mg/kg bw/d) and is known as the acceptable daily intake.

Preclinical safety assessments are considered before designing appropriate clinical studies. Those clinical studies followed traditional protocols approved by an institutional review board. For instance, in the recent inclusion of oligosaccharides, such as 2’-fuctosyllactose, lacto-N-neotetraose, fructooligosaccharides, and galactooligosaccharides, the safety of these substances was reviewed by GRAS panel members whose training and experience qualified them as experts. The GRAS documentation for these substances is available at the FDA GRAS Inventory website [14].

During the past several decades, many new ingredients, such as probiotics and docosahexaenoic acid, have been introduced into infant formulas marketed in the United States. Although some infant formula components are not listed in the Infant Formula Act, including lactoferrin, PUFAs, probiotics, and oligosaccharides, they are now commonly found in infant formulas in an attempt to mimic the health benefits of human milk. The United States regulations have not established minimum or maximum values for these components.

## Impact of Environmental Chemicals on Infant Development

Chemicals in the environment can have lasting and detrimental effects on infants and young children, because these individuals are still developing and are therefore more susceptible to known and unknown pathways of toxicity than adults. This susceptibility can lead to alterations in cognitive performance, delays in puberty and behavioral development, and adverse effects on the immune system. Major factors accounting for this early life-stage susceptibility include: allometric scaling [higher food (formula) to body weight consumption ratio]; immature metabolic capacity for chemical detoxification and excretion (allows greater distribution of chemicals to different organ systems); and immaturity of the blood–brain barrier (allows chemical access to the developing brain) [15].

Environmental contaminants of particular concern for infantile and early life exposure include heavy metals such as lead, mercury, arsenic, and cadmium [[16], [17], [18]], plasticizers such as phthalates [19], “forever chemicals” such as perfluoroalkyl and polyfluoroalkyl substances [20], microplastics, and nanoplastics [21], endocrine-disrupting chemicals such as polychlorinated biphenyls, dioxins, some pesticides, and flame retardants [22,23], and some food additives and dyes classified either as direct (e.g., colorants, flavoring agents, and preservatives) or indirect (based on processing equipment that includes adhesive, dyes and coatings, and packaging) [24]. Given that infants not fed on human milk rely primarily on formula as their primary nutritional source, they may experience a greater risk from these environmental contaminants.

Consumer Reports recently tested 41 types of powdered formula for several toxic chemicals, including heavy metals, and published their findings on 18 March, 2025 [25]. Mercury and cadmium were not detected or were well below levels of concern in the infant formulas tested; however, concern remained over the detectable concentrations of arsenic and lead. The risks and effects of these contaminants through formula depend on the levels and duration of exposure, as well as whether the infant may have already been exposed prenatally through the intrauterine environment and during a vulnerable stage of development. Here, we review evidence as to: *1*) which environmental contaminants (focusing on heavy metals as examples) may be most beneficial to test for; *2*) summarize what existing recommendations and guidelines have been established internationally for environmental contaminants; and *3*) briefly identify research gaps in scientific understanding of environmental contaminants in infant formula.

### Heavy metals

Arsenic originates in the earth’s crust but can contaminate groundwater or other ingredients during the manufacturing or preparation of infant formula. Inorganic arsenic is a known carcinogenic hazard. The Consumer Report [25] calculated a “hazard quotient” above the level below which no adverse health effects would be expected to occur (assuming a 3-mo-old infant of average size eating an average amount each day). Although most of the formulas tested were below the hazard quotient for arsenic, only 2 approached the limit, which may warrant further testing.

Lead is a pervasive environmental contaminant that has been linked to adverse effects on neurodevelopment. Because it is challenging to render food or water altogether void of lead, the FDA’s oversight plan “Closer to Zero” sets permissible limits under 10 or 20 parts per billion (ppb) bracketing the United States Environmental Protection Agency (EPA) action if triggered by lead concentrations >1 ppb in public water [26]. Notably, the Closer to Zero guidance is specified for infant foods and does not include infant formula. Consumer Reports found lead concentrations ranging from 1.2 to 4.2 ppb in most powdered formulas, thereby falling below the Closer to Zero goal. Mixing powdered formula with tap water that contains lead could increase daily exposure above the permissible threshold, underscoring the need for broad consumer education about the many environmental sources of lead.

### International guidelines

In the United States, there are no established maximum allowable levels set for any environmental contaminants in infant formula. Although the FDA’s Closer to Zero Initiative, launched in 2021, aims to develop action levels for heavy metals in juices and foods intended for infants and children, particularly in food where some contamination is unavoidable, infant formula has not been included. Furthermore, no clear consequences or enforceable regulations have been established yet for any contaminants that exceed the provisional action levels [26]. Although many formula manufacturers in the United States may already conduct independent testing for environmental pollutants, such as heavy metals, it is essential to standardize testing practices across the industry as well as an established list of which environmental contaminants are most relevant to assess. Evaluation should include both individual ingredients and finished products [27]. Additionally, increased transparency from the United States FDA regarding the frequency of testing and the most recent contaminant levels is critical for rebuilding consumer trust in the safety of United States formulas.

For several specific environmental contaminants, maximum levels have already been established for infant formula in the European Union (EU), Canada, and Australia/New Zealand; however, even among our international counterparts, there is variation on which environmental contaminants to monitor (Table 2). The EU maintains the most extensive maximum allowable limits for infant formula contaminants, including lead, cadmium, arsenic, tin, because it is an antirust barrier between the steel and the epoxy lining of canned infant formulas, polycyclic aromatic hydrocarbons, and melamine, with updates implemented by the European Commission [28]. By comparison, Australia has proposed maximum limits for only 4 infant formula contaminants (lead, vinyl chloride, aluminum, and acrylonitrile) in its own 2023 updated review on the regulation of infant formula products revised under Proposal P1028—Infant Formula [29]. For Canada, maximum limits are proposed for only lead (0.01 ppm) in infant formula [30]. Codex Alimentarius (Codex) an international food standards body established by the FAO and WHO, of which the United States is a member, provides another reference with recently updated contaminant specifications in infant formulas (for lead and melamine) [31].

**TABLE 2:** Summary of international guidelines for infant formula contaminants.

United States	Canada	Australia/New Zealand	European Union
Lead (0.01 ppm for infant foods, no level for formula)^1^	Lead (0.01 ppm for formula)	Lead (0.02 mg/kg for formula)	Lead (0.01 mg/kg for liquid formula, 0.02 mg/kg for powder)
Cadmium (action levels pending for infant food, not formula)^1^		Tin (250 mg/kg, canned food)	Tin (50 mg/kg, canned formula)
Arsenic (action levels pending for infant food, not formula)^1^		Vinyl chloride(0.01 mg/kg)	Cadmium (0.01 mg/kg for liquid formula, 0.005 mg/kg for powder)
		Aluminum (0.05 mg/100 ml)	Arsenic (0.01 mg/kg for liquid formula, 0.020 mg/kg for powder)
		Acrylonitrile (0.02 mg/kg)	Polycyclic aromatic hydrocarbons (1 μg/kg)
			Melamine (1 mg/kg, 0.15 mg/kg)
			Aflatoxin M1 (0.025 μg/kg MRL)

Abbreviation: MRL, maximum residual limit.

1 United States has no current limits set for formula contaminants, but we included some of the heavy metal contaminants that are being studied under FDA’s closer to 0.

Another contaminant class of concern is the mycotoxins. Maximum residual limits (MRLs) for mycotoxins vary significantly depending on the specific mycotoxin, the commodity (e.g., grains, nuts, milk), and the regulatory body (e.g., the United States FDA, European Commission, and Codex Alimentarius Commission). MRL generally range from <10 to >500 ppb. The Codex Alimentarius Commission and the EU have set the strictest MRL for aflatoxin M1 (AFM1), the primary aflatoxin found in milk, for infant formula at 0.025 μg/kg (0.025 ppb). This limit is lower than the limits in the United States (0.5 ppb) for milk, but the Codex/EU limits are specifically for infant formula because of infants’ higher susceptibility because of their lower body weight, underdeveloped detoxification systems, and more restricted diet. The MRL for aflatoxin B1 (AFB1) is generally set for solid foods, whereas a separate limit is set for AFM1 in liquid infant formula. Although there are no explicit limits for AFB1 in infant formula, international guidelines and regulations aim to keep total aflatoxin concentrations <0.1 μg/kg (0.1 ppb) dry matter in infant and infant foods to protect this vulnerable subpopulation [28].

Regardless of the initial selection of regulated environmental contaminants in infant formula, a systematic review cycle should be conducted at least every 5 y with an established expert panel, allowing for an expanding list as research identifies additional harmful substances.

Although there has been research conducted in mouse models as well as in cross-sectional population-based studies, the health effects of mild-moderate heavy metal exposure in infants are relatively understudied. Toxic metals do not exist in isolation and can often disrupt the homeostasis of essential elements [32]. Thus, additional studies are needed to examine whether exposure to a mixture of contaminants could have synergistic effects on one another and potentially interact with some of the newly added bioactive ingredients. Understanding these interactions may help guide the establishment of maximum allowable levels that have not yet been determined and established.

As mentioned previously, although human milk is the ideal source of nutrition with multiple immunologic benefits, infants can also be exposed to environmental contaminants, specifically heavy metals, through the maternal environment and diet [33]; consequently, effective reduction of ecologic contaminants for all infants, regardless of diet, requires not only regular monitoring systems, but also a collaboration with the EPA to ensure that environmental regulations stay in place to safeguard our food supply from industrial heavy metal pollution through soil, air, and water pathways.

## New Approach Methods for Basic Research and Discovery in Risk Science

Toxicology testing plays a crucial role in ensuring the safety of regulated products and is typically conducted in animal studies. New approach methods (NAMs) are being developed for toxicity testing to enhance the capacity to quickly and accurately predict the potential risk from chemical, physical, or biological agents in the animal-free zone, leveraging the latest advances in science and technology, such as organs-on-chip or mathematical modeling.

The EPA defines a NAM as “any technology, methodology, approach, or combination thereof that can be used to provide information on chemical hazard and risk assessment that avoids the use of intact animals” [34]. NAMs are part of the FDA’s Predictive Toxicology Roadmap to support replacement, reduction, and refinement of animal studies” [35].

A shared vision for the future of toxicity testing in the 21st century was formalized in a federal collaboration among the NIH, EPA, and FDA [36]. The overarching goal is to screen large numbers of chemicals for the potential to disrupt cellular processes underlying adverse health effects using high-throughput robotics and computer modeling. Multiple modalities contribute to this effort with information and insights from NAMs playing a critical role in translating research into better healthcare strategies, including use of high-throughput screening for ascertaining bioactivity profiles of chemical–biomolecular target interactions assayed across an in vitro concentration range; high-throughput kinetics as an in silico tool for in vitro to in vivo extrapolation of dosimetry across species and life-stage; structure-activity relationships to as a computational tool to infer/predict bioactivity potential for untested chemicals having structural similarity to tested chemicals; microphysiological systems to directly test chemical effects using human cell-based organotypic culture models; adverse outcome pathways for evidence-based elucidation of toxicity pathways from molecular initiating event to adverse outcome of regulatory value in risk science; and agent-based modeling for reconstructing cellular dynamics in silico using computer models harboring self-organizing intelligence based on known biology [37].

## Safety by Design

Throughout this article, numerous aspects of the safety of infant formula ingredients and the finished products have been presented. Although potential environmental contaminants are briefly discussed in this article, the extensive list of classic mycotoxins and endotoxins, along with their respective maximum levels in infant formula, has not been established in the United States or by the Codex. However, GRAS dossiers for infant formula ingredients typically address this issue by leveraging upper limits, tolerable intake levels, and margins of exposure, using WHO guidelines for a range of pesticides. The EU has established maximum residue levels for mycotoxins, heavy metals, and pesticides [28]. A typical surrogate indicator of mycotoxin exposure is AFB1 at 15 ppb; however, neither Codex, the EU, nor the United States has codified an action level of mycotoxins in infant formula per se.

Potential microbial contamination standards have been reactionary in the United States. Regardless, the FDA has established strict microbial limits for infant formula. Of primary concern for the agency are Salmonella (absent in 25 g), Cronobacter (absent in 10 g), coliforms (maximum not specified), Shiga-toxin–producing *Escherichia coli* (maximum limit not established), and *Bacillus cereus* (maximum limit not established)*. Staphylococcus aureus* (not detected in 1 g) and *Pseudomonas aeruginosa* (not detected in 1 g) are typically included in the product specifications. The limit for *B. cereus* is not explicitly stated in FDA regulations, but they do require that *B. cereus* must not be detected in infant formula. This organism continues to receive attention because of its production of an emetic toxin and ≤5 different enterotoxins, the assessment of which is not specified for infant formula ingredients or the finished product [38].

*Enterobacter sakazakii*, now known as *Cronobacter sakazakii*, an environmental contaminant, is a serious source of infections in infants. This organism forms a biofilm, which can become problematic on the surfaces of food production equipment and facility structures. These biofilms are difficult to eliminate because of their resistance to typical cleaning and sterilization procedures [39].

The FDA has not established maximum levels for yeast and molds and has yet to address viruses that may pose a public health risk. Fortunately, thermal processing conditions for producing powder and liquid forms of infant formula can inactivate most viruses and other microbes. Although microbial contamination risks persist, the FDA’s safety standards for infant formula ingredients and quality control procedures are designed to reduce these risks; however, as discussed above, implementation challenges and industrial variability are limiting their effectiveness [40].

In conclusion, the safety, regulation, and availability of infant formula are vital public health concerns that demand coordinated oversight, scientific rigor, and more well-defined guidelines (Table 3). The 2022 formula shortage exposed critical vulnerabilities in the United States regulatory framework, prompting initiatives such as Operation Stork Speed to reevaluate and modernize policies. This article underscores the need for streamlined FDA approval processes, enhanced transparency in ingredient safety through GRAS and food additive pathways, and stronger protections and guidelines on levels of microbials as well as environmental contaminants, such as heavy metals. We have highlighted advances in preclinical diagnostic tools, such as NAMs, that can supplant animal testing with human cell-based in vitro assays and in silico models, which present as a possible resource for more predictive and efficient safety assessments. Moving forward, harmonizing our standards and methods required to evaluate and incorporate new infant formulas into our market in alignment with international guidelines will be key to safeguarding the nutrition of the nation’s most vulnerable population.

**TABLE 3:** Key summary points.

Regulatory reform	Operation stork speed launched to modernize infant formula oversight after 2022 shortages. Current FDA processes are slow and complex, making it difficult for new formulas to enter the market.
Ingredient safety	GRAS and food additive pathways require safety data, but food additive petitions require more safety information and cannot be marketed until FDA approval is granted.
Contaminant risks	Heavy metals, PFAS, and other toxins can be found in formulas and infants can be at increased risk of effects. United States lacks enforceable limits, unlike EU, Canadian and Australian counterparts.
Testing innovations	NAMS may be a helpful nonanimal toxicology method.
Call for action	Legislative updates, supply chain transparency, and alignment with global safety standards are needed.

Abbreviations: EU, European Union; FDA, Food and Drug Administration; GRAS, generally recognized as safe; NAMS, new approach methods; PFAS, perfluoroalkyl and polyfluoroalkyl substances.

## Author contributions

The authors’ responsibilities were as follows – SAA, JTB, VCC, ND, AG, MIG, AG, JAK, TBK, SK, TS: participated in the conference described and contributed to the preparation of the manuscript; and all authors: read and approved the final manuscript.

## Funding

JTB has received honoraria or travel funds from the Global Organization for EPA and DHA, the global dairy platform, Danone, and research support from the National Cattlemen’s Beef Association. MIG has previously served as a scientific advisor for Begin Health and Bobbi. VC has served on the Speaker’s Bureau for Nutricia and Abbott Nutrition. The other authors have no additional financial support to declare.

## Conflict of interest

SAA is editor-in-chief of Advances in Nutrition and played no role in the Journal’s evaluation of the manuscript. All other authors report no conflicts of interest.
